# Revolving Door in Older Patients: An Observational Study of Risk Assessment of Rehospitalization Using the BRASS Scale

**DOI:** 10.3390/diseases13100325

**Published:** 2025-10-01

**Authors:** Francesco Saverio Ragusa, Anna La Vattiata, Antonio Terranova, Giuseppina Pesco, Davide Mariani, Ligia J. Dominguez, Nicola Veronese, Pasquale Mansueto, Mario Barbagallo

**Affiliations:** 1 Geriatric Unit, Department of Internal Medicine and Geriatrics, University of Palermo, 90133 Palermo, Italy; pasquale.mansueto@unipa.it (P.M.); mario.barbagallo@unipa.it (M.B.); 2 Unità Operativa Complessa Medicina, Ospedale Buccheri La Ferla, 90123 Palermo, Italy; anna.lavattiata15@gmail.com; 3 Department of Health Promotion, Maternal-Infant, Internal Medicine and Specialization of Excellence “G. D’Alessandro”, University of Palermo, 90133 Palermo, Italy; antonio.terranova01@unipa.it; 4 Integrated Medical Activities Department, University Hospital “P. Giaccone” Hospital, 90133 Palermo, Italy; giuseppina.pesco@policlinico.pa.it (G.P.); davide.mariani@policlinico.pa.it (D.M.); 5 Department of Medicine and Surgery, Kore University of Enna, 94100 Enna, Italy; ligia.dominguez@unikore.it; 6 Faculty of Medicine, Saint Camillus International University of Health Sciences, 00131 Rome, Italy; nicola.veronese@unicamillus.org

**Keywords:** older people, revolving door, hospitalization, mortality

## Abstract

**Introduction**: The “revolving” door is a phenomenon that refers to the rehospitalization of older patients who, after being discharged, soon require specialized hospital care again. Unfortunately, the use of tools able to predict this phenomenon is still limited. The aim of this study was to highlight the validity of the Blaylock Risk Assessment Screening (BRASS) Scale in objectively assessing the risk of rehospitalization and mortality among older patients. **Methods**: Patients were classified as low, medium, or high risk using the BRASS scale. Adverse events (rehospitalization or death) were recorded at baseline and at 12 months. Kaplan–Meier curves evaluated survival and rehospitalization across risk groups, and ROC analysis assessed the BRASS Scale’s predictive value for mortality. **Results**: Out of 179 enrolled older adults (mean age 67.7 years), 54.2% were classified as low risk, 29.5% as medium, and 16.8% as high risk based on the BRASS Scale. High-risk patients had significantly higher mortality (HR: 4.40; 95% CI: 1.60–12.19, *p* = 0.004) and lower survival rates, while intermediate-risk patients had increased rehospitalization (HR: 2.11; 95% CI: 1.09–4.08, *p* = 0.02). The BRASS scale showed good predictive value for mortality (AUC 0.76). **Conclusion**: The BRASS Scale has a good predictive value for negative outcomes, and it confirms that a substantial proportion of older patients are at risk of future hospital readmissions and complex discharges. These findings underscore the importance of early post-discharge care planning and the implementation of protected discharge programs.

## 1. Introduction

The aging of the population, now a global phenomenon, is a complex demographic process involving economic, physical, and cultural aspects. According to data from the Italian National Institute of Statistics, as of 1 January 2024, there were 14 million people in Italy older than 65 years old, and the natural balance, that is, the difference between births and deaths, remained negative in 2023, with 379,339 new births and 660,600 deaths, reflecting a trend common to many countries in recent times [1].

Among this population, frailty is a very common condition, representing a highly complex condition that encompasses multiple definitions. It can be described as a multidimensional geriatric syndrome, a clinically recognizable state of increased vulnerability resulting from aging-associated decline in reserve and function across multiple physiologic systems, such that the ability to cope with every day or acute stressors is comprised [2].

The pathological and psychological profile characterizing this type of patient is highly specific: it refers to individuals over the age of 65 suffering from multiple comorbidities that, in turn, define a state of frailty, exposing them to an urgent and continuous need for both inpatient and outpatient care [3,4]. The aging population and the lack of adequate community-based care have led this study to focus on the phenomenon of the “revolving door”, which refers to the rehospitalization of older patients who, after being discharged, soon require specialized hospital care again [5]. This condition causes both psychological and physiological stress for the patient and places a financial and economic burden on the healthcare system [6].

Several tools can assist medical and nursing staff in identifying patients at risk of the “revolving door” phenomenon. One such tool is the Blaylock Risk Assessment Screening Score Index, also known as the BRASS Scale. This assessment index was introduced in the fields of geriatrics and gerontology to provide more specific and selective data on the condition and integrated care of elderly patients over the age of 65 [7,8]. Its purpose is to predict the risk of prolonged hospitalization and complex discharge, enabling the activation of an extra-hospital care network for patient management [7].

A previous Italian study, involving 122 participants, aimed to analyse how the BRASS Scale could assist clinicians and nurses in preventing the “revolving door” phenomenon in older patients [9]. The study found a positive association between the BRASS Scale and rehospitalization in older individuals. However, this association was not statistically significant. Additionally, nearly 50% of the participants were from the cardio-surgical department, leading to a higher prevalence of frailty, as those undergoing such procedures are typically more vulnerable [9]. Another Spanish study of 370 patients revealed how higher BRASS Scale score was strongly associated with a greater risk of mortality. Patients requiring continued care had higher average BRASS scores compared to those who did not; however, the “revolving door” phenomenon was not considered, as there was no actual follow-up on subsequent hospitalizations [10].

Older adults represent a population at particularly high risk of rehospitalization and mortality, especially in the context of prolonged care needs. Identifying reliable tools to assess this risk objectively is therefore essential for improving patient outcomes and guiding appropriate interventions. Among the available instruments, the BRASS Scale has gained attention as a potential tool for risk stratification in older patients, although further research is needed to confirm its validity in this setting. The aim of this study is to highlight the validity of the BRASS Scale in objectively assessing the risk of rehospitalization and mortality among older patients, implementing targeted interventions to mitigate this phenomenon.

## 2. Materials and Methods

### 2.1. Study Populations

This study was conducted using data collected from the Internal Medicine and Geriatrics Ward at the University Hospital “Policlinico Paolo Giaccone” in Palermo, Italy, between July 2023 and September 2024. Ethical approval was granted by the local ethics committee “Comitato Etico Locale Palermo 1” of the same hospital (n. 07/2023; approval date: 12 December 2023). Written informed consent was obtained from all participants. For the purposes of this research, we included only older adults, specifically, men and women aged over 65 years, who were hospitalized in our department. Individuals were excluded if they were under 65 years of age or if they were unable to comprehend or provide informed consent.

### 2.2. Exposure: BRASS Scale

The BRASS Scale was administered on the spot within the first three days of hospitalization, consisting of 10 items designed to assess the functional, social, and psychological status of the patient [8]. Its main goal is to identify older patients, aged 65 and over, who are at risk of experiencing a “difficult discharge”, i.e., patients whose post-hospital condition is so complex or moderately complex to require careful planning and organization of the discharge process.

Supplementary Table S1 shows how the BRASS Scale consists of 10 items: age, living conditions and social support, functional status, cognitive status, behavioral model, mobility, sensory deficits, number of previous hospitalizations/emergency room visits, number of active clinical problems, and number of medications taken. Each item is divided into different parameters, with each parameter assigned an increasing numerical score directly proportional to the patient’s baseline condition. As the patient’s disabilities and frailties increase, the numerical score rises (for example, Item 7 refers to sensory deficits and includes three parameters: 0 = none, 1 = visual or auditory deficits, 2 = both, visual and auditory deficits).

Once the scale is completed, the scores from all items are summed, and the total score determines three risk categories [11]:

Low risk (score 0–10): patients with a low risk of requiring protected discharge, for whom no individualized post-hospital care planning is necessary; at low risk of hospital readmission.

Medium risk (score 11–19): patients with a moderate risk of requiring protected discharge, necessitating individualized post-hospital care planning due to possible complex clinical conditions; at moderate risk of hospital readmission.

High risk (score ≥ 20): patients with a high risk of requiring protected discharge, with significant medical or social issues necessitating the establishment of a continuity of care plan; at high risk of hospital readmission.

### 2.3. Outcomes: Rehospitalization, Mortality

Rehospitalization was detected with a phone call after 12 months: participants were asked whether they had experienced a second hospitalization, the hospital department in which the admission occurred, and the major diagnosis of their rehospitalization. Mortality was also assessed during first hospitalization by asking the caregivers, in the event of the patient’s death, for the date on which it occurred.

### 2.4. Participant Characteristics

The following factors were considered as potentially important covariates, based on the literature [12]: sex, social status (family, institute, and living alone), cause of hospitalization, cause of second hospitalization and death, treated as categorical variables. Age was considered as a continuous variable.

### 2.5. Statistical Analysis

Quantitative data were summarized using means and standard deviations (SDs), while categorical data were presented as frequencies and percentages. Baseline characteristics of study participants were compared across categories determined by the BRASS Scale. For categorical variables, Chi-square tests were employed, or Fisher’s exact test was used when expected cell counts were below 5. For continuous variables, one-way ANOVA was conducted, followed by Bonferroni post hoc analysis to account for multiple comparisons.

Kaplan–Meier survival curves were utilized to explore patient survival and rehospitalization across the three groups, categorized by risk according to the BRASS Scale. A Cox proportional hazards model was used to assess the impact of BRASS Scale on negative outcomes, such as mortality and rehospitalization. Hazard ratios (HRs) and 95% confidence intervals (95% CIs) were calculated. Univariate analysis was performed first, followed by a multivariate model including significant predictors and adjustment variables (age, sex). A *p*-value threshold of 0.10 was used to select variables, and collinearity was checked using the Variance Inflation Factor (VIF), excluding variables with VIF >2, except adjustment variables. The ROC curve for the BRASS Scale was generated to assess its predictive value for mortality, and the Area Under the Curve (AUC) was also calculated. A BRASS Scale score of 0–10 was considered low risk, 11–19 as medium risk, and ≥20 as high risk.

All statistical tests were two-tailed, and a *p*-value < 0.05 was considered to be statistically significant. All analyses were performed using RStudio, Version 2024. 12.+1 563.

## 3. Results

Out of the 198 patients initially screened, 179 older adults (mean age 67.7 years, 49.7% female) were enrolled in the study. The remaining 19 individuals were excluded due to severe dementia or the inability to provide informed consent, with no caregiver available to assist, or due to loss to follow-up.

Based on the BRASS Scale score, 97 patients (54.2%) were classified as low risk, 30 (29.5%) as medium risk, and 52 (16.8%) as high risk. Patients in the high-risk BRASS group were significantly older (*p* < 0.001), more likely to live alone (*p* = 0.01), and experienced higher rates of rehospitalization (*p* = 0.004) and mortality (*p* < 0.001) compared to those in the lower-risk groups (Table 1).

According to the multivariate analysis for mortality (Table 2), a statistically significant increase in mortality was observed only in the high-risk group compared to the low-risk group (HR 4.40, 95% CI 1.59–12.18, *p* = 0.004); 74 patients (41.3%) were readmitted, showing that nearly half of the patients analysed experienced a new hospitalization within the following 12 months.

Regarding rehospitalization (Table 3), the intermediate-risk group demonstrated a significantly higher risk of rehospitalization relative to the low-risk group (HR 2.11, 95% CI 1.09–4.08, *p* = 0.02). No significant associations were identified for either age or sex in these analyses.

**Figure 1:**
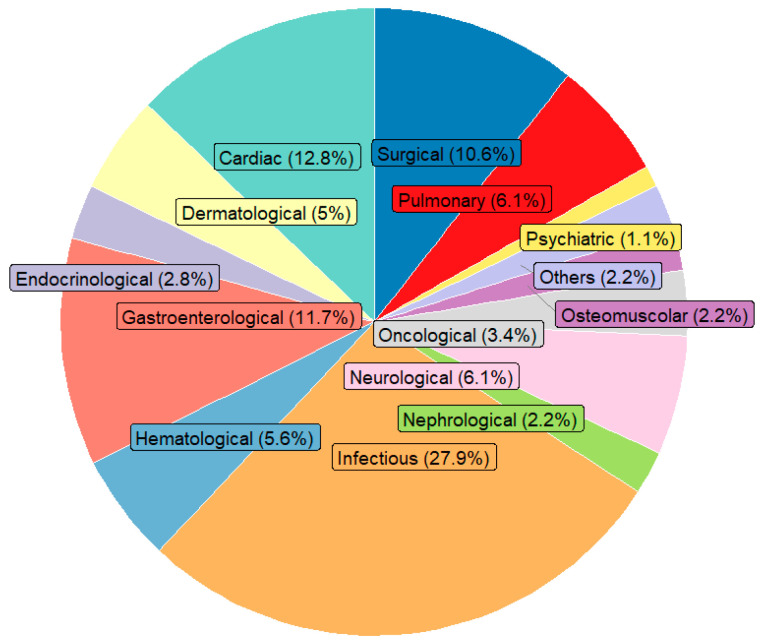
Distribution of hospitalization by medical category. Note: the sum of percentage may not equal 100% due to rounding.

**Figure 2:**
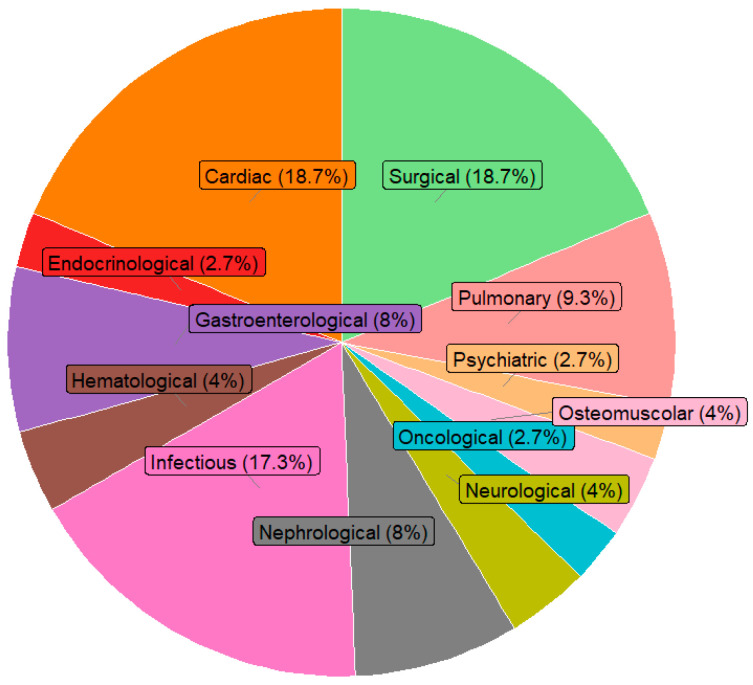
Distribution of rehospitalization by medical category. Note: the sum of percentage may not equal 100% due to rounding.

**Figure 3:**
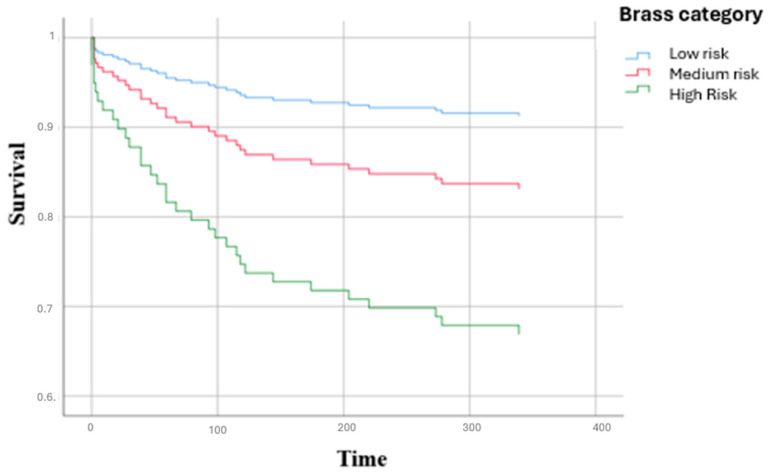
Survival curves of participants according to BRASS Scale.

**Figure 4:**
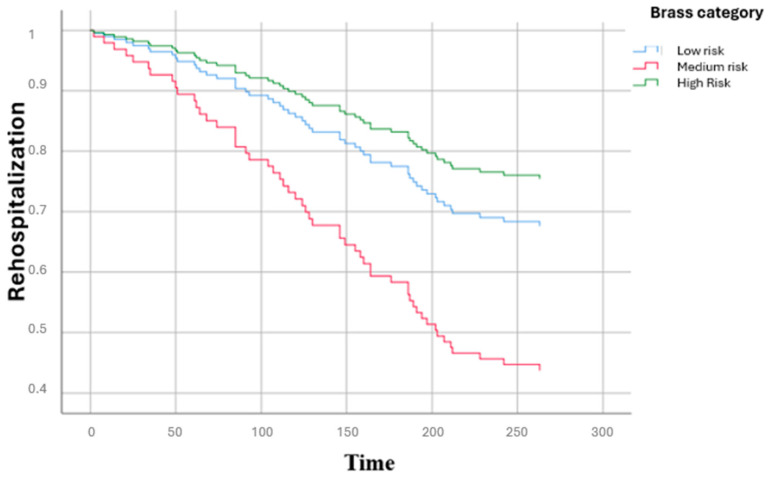
Rehospitalization curves of participants according to BRASS Scale.

Figure 1 shows the distribution of the main causes of hospitalization by clinical area: infectious diseases represented the first cause (27.9%), followed by cardiac diseases (12.8%), gastroenterological diseases (11.7%) and surgical causes (10.6%). Figure 2 illustrates the main causes of rehospitalization by clinical area: in this picture surgical causes and cardiac diseases became the main reason (both 18.7%) and then infectious disease (17.3%), pulmonary disease (9.7%) and nephrological and gastroenterological diseases (8% both), showing how there was a great decrease in infectious diseases (−14.2%) and an important increase in surgical causes (+8.1%) and cardiac diseases (+5.9%). Figure 3 presents the Kaplan–Meier survival curves stratified according to BRASS risk categories. As shown, patients classified in the high-risk group experienced a markedly lower probability of survival over the entire follow-up period compared to those in the medium- and low-risk categories. The survival probability for high-risk patients declined steeply within the first months and remained consistently below that of the other groups throughout the observation period. In contrast, the low-risk group demonstrated the most favorable survival outcomes, with survival probabilities remaining consistently high and stable over time, while the medium-risk group exhibited intermediate survival patterns, falling between the two extremes. The differences among the survival curves were statistically significant according to the log-rank test (low vs. high risk: *p* < 0.0001; intermediate vs. high risk: *p* < 0.001; intermediate vs. low risk: *p* = 0.82). Figure 4 depicts the Kaplan–Meier curves for rehospitalization events across the same BRASS risk categories. Interestingly, in this analysis, the intermediate-risk group showed a significantly greater probability of rehospitalization compared to both the low- and high-risk groups throughout the follow-up period. The low-risk group maintained the most favorable trajectory, with the lowest cumulative risk of rehospitalization over time. The unexpectedly higher rehospitalization rate in the intermediate-risk group, relative even to the high-risk group, may indicate distinct clinical or social factors influencing rehospitalization risk beyond those captured by the BRASS score alone, warranting further investigation. Here too, the differences between curves were statistically significant (low vs. high risk: *p* < 0.0001; intermediate vs. high risk: *p* < 0.001; intermediate vs. low risk: *p* = 0.52).

**Figure 5:**
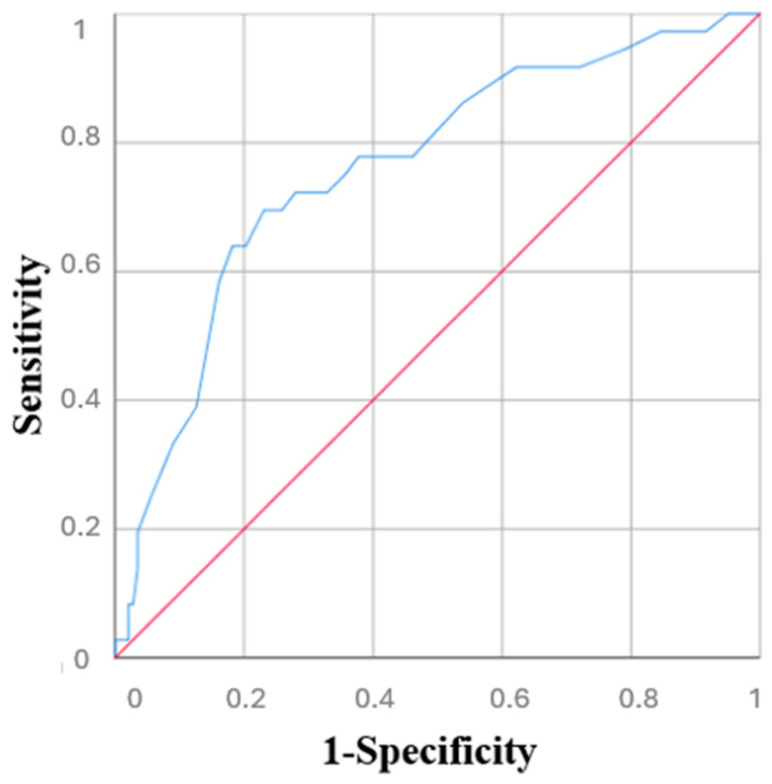
ROC curve of BRASS Scale’s predictive value for mortality.

Figure 5 shows the ROC curve for the BRASS Scale’s predictive value for mortality. The ROC curve analysis demonstrated that this index had a good predictive value for mortality (AUC = 0.76, 95% CI 0.67–0.85).

## 4. Discussion

This study represents one of the pioneering efforts aimed at emphasizing the usefulness and validity of the BRASS Scale as an objective tool for assessing the risk of rehospitalization among older patients. The primary aim of this study was to evaluate the effectiveness of the BRASS Scale in objectively identifying older patients at risk of difficult discharge and hospital readmission to support the implementation of targeted post-discharge care strategies and reduce the incidence of the “revolving door” phenomenon among frail older adults. Most of the previous studies underlined the importance of BRASS Scale only for detecting inpatients requiring a specific and adapted discharge planning; in contrast, our study evaluated the occurrence of readmission and survival over time. The BRASS Index is widely used by nurses around the world as a standardized tool to assess patient complexity and discharge needs. Its simplicity, reliability, and clinical relevance have made it a globally adopted instrument in hospitals and healthcare systems for guiding early discharge planning [10].

Among the 179 patients included, we found 29.5% at medium risk, 54.2% at low risk and 16.8% at high risk according to the BRASS Scale. Thus, the largest portion of participants were at medium risk of difficult discharge and consequently at risk of rehospitalization. This condition underscores the clinical and psychological distress faced by many older patients and highlights the need for targeted post-discharge care to prevent the “revolving door” phenomenon [13].

Although patients in the high-risk group exhibited higher mortality rates, they were not readmitted more often than those in the medium-risk group—a finding that is not immediately intuitive and deserves further discussion. One possible explanation is a clinical tendency to avoid rehospitalizing the most frail patients, possibly due to ageism or family preferences to forgo hospitalization in favor of home-based palliative care for those in severely compromised conditions. This highlights the need to carefully consider care strategies and hospitalization criteria in the management of highly vulnerable patients. In the multivariate analyses, age was not statistically significant, although it showed an upward trend. This result highlights a key point: while age is often considered a primary risk factor, it alone does not capture the complexity of older patients’ health status. Frailty assessment offers a more comprehensive evaluation, incorporating functional, cognitive, and social dimensions that are not reflected by age alone. Previous studies have similarly demonstrated that frailty indices outperform chronological age in predicting adverse outcomes, including mortality and rehospitalization [4,14]. Our findings therefore support the growing consensus that clinical decision-making in older adults should be guided by frailty assessment rather than age alone. The lack of statistical significance for age further supports the need to use frailty assessment tools, rather than relying solely on chronological age, when stratifying risk and planning care.

Consistent with our findings, older patients at medium risk are most likely to be rehospitalized shortly after discharge. A previous Italian study of 428 participants revealed that patients with a greater BRASS score experienced longer hospitalization [15]. While the BRASS Scale score closely reflected illness severity, no follow-up data on outcomes like mortality or rehospitalization were collected. Furthermore, the similarity between the conditions leading to readmission and those from the initial hospitalization highlights important concerns about the quality of post-discharge care for older patients.

There are not only clinical reasons behind the frequent rehospitalizations of older people; in fact, racial and socioeconomic differences in health care outcomes and quality indicators, like 30-day hospital readmission rates, are well established and need urgent attention to ensure fair and high-quality care for every patient [16]. An American study analysed 1,508,402 surgical admissions between 2007 and 2010 and found that Black individuals had a 19% higher probability of being readmitted after surgery compared to white individuals and this risk was 34% higher when the care was provided at hospitals that primarily serve minority populations [17].

Our study also showed that, over time, high-risk patients had significantly lower survival rates than those in the medium- and low-risk groups, with the low-risk group consistently showing the best survival outcomes throughout the follow-up period. This represents a contribution to the existent literature, as a previous French study involving 110 non-neurological ICU patients found no independent association between BRASS score and mortality [18]. However, in that study, patients were sedated with propofol, and the care setting was more complex than that of an internal medicine ward.

In the present study, we also found that the BRASS Scale reliably predicts mortality, effectively identifying patients at higher risk of death during hospitalization and after discharge, reinforcing its value as a tool for early risk stratification and discharge planning.

A previous Dutch study involving 503 participants found that BRASS Scale was a good predictor, specifically for identifying patients unlikely to be discharged at home, highlighting its strong correlation with post-discharge challenges [19]. However, they analysed only physical outcomes, such as personal care, housekeeping and mobility, without data about mortality.

The present study should be interpreted within its strengths and limitations. As previously said, this study is among the first to highlight the usefulness and predictive value of the BRASS Scale not only in identifying patients in need of specific discharge planning, but also in predicting adverse outcomes, including post-discharge mortality. Notably, it provides new evidence of the scale’s strong predictive value for mortality, extending its applicability beyond discharge planning and offering insights not explored in previous studies. Secondly, unlike many previous studies that relied solely on cross-sectional data, this study included a follow-up period to assess post-discharge outcomes, providing a more comprehensive understanding of patient trajectories and risks. This study also has several limitations. Firstly, the study was conducted in a single hospital with a relatively small sample, which may limit the generalizability of the findings to other settings or larger, more diverse populations. Secondly, the retrospective nature of the analysis might introduce biases related to data collection and completeness, compared to a prospective study design. Finally, recording data about rehospitalization and mortality using self-reported data may introduce a recall bias.

Although several newer risk prediction models for hospital readmission have been proposed [20], incorporating medical comorbidities, prior utilization, functional status, and social determinants of health, the BRASS Index remains widely used in clinical practice for discharge planning, particularly in older populations. Our study aimed to validate the BRASS Index in our specific setting, demonstrating that it continues to stratify patients effectively according to risk of readmission and mortality. We acknowledge that the identification of actionable interventions to reduce readmission risk represents the next critical step; however, establishing the validity and utility of existing tools is essential before such interventions can be meaningfully designed and implemented. Our findings therefore provide a foundation for future studies aimed at linking risk stratification with targeted strategies to improve patient outcomes.

## 5. Conclusions

In conclusion, the full BRASS Scale scoring, already considered a reliable tool for assessing complexity and discharge needs, demonstrates that a large portion of older patients are at risk of future hospital readmissions and difficult discharges. This highlights the need for careful planning of post-hospital care and for implementing protected discharge programs in collaboration with general practitioners, with the goal of early intervention and increasing prevention of the “revolving door” phenomenon.

## Figures and Tables

**Table 1:** Characteristics of participants.

Variables	High (N = 52)	Medium (N = 30)	Low (N = 97)	Overall (N = 179)	*p* Values
Age					*p* < 0.001
Mean (SD)	79.8 (7.86)	71.2 (12.5)	60.2 (15.5)	67.7 (15.7)	
Sex					*p* = 0.03
Female	26 (50.0%)	21 (70.0%)	42 (43.3%)	89 (49.7%)	
Social					*p* = 0.01
Family	32 (61.5%)	19 (63.3%)	80 (82.4%)	131 (73.3%)	
Institution	7 (13.5%)	2 (6.7%)	2 (2.1%)	11 (6.1%)	
Alone	13 (25.0%)	9 (30.0%)	16 (16.5%)	38 (21.2%)	
Rehospitalization					*p* = 0.004
No	37 (71.2%)	10 (33.3%)	58 (59.8%)	105 (58.7%)	
Yes	15 (28.8%)	20 (66.7%)	39 (40.2%)	74 (41.3%)	
Death					*p* < 0.001
No	29 (55.8%)	25 (83.3%)	89 (91.8%)	143 (79.9%)	
Yes	23 (44.2%)	5 (16.6%)	8 (8.2%)	35 (19.6%)	

**Table 2:** Risk for mortality for factors included in the multivariate analysis.

	HR	95% CI Lower	95% CI Upper	*p*-Value
Low Risk (reference)				
Intermediate Risk	2.02	0.62	6.53	0.23
High Risk	4.40	1.59	12.18	0.004
Age	1.01	0.98	1.05	0.31
Gender	0.80	0.56	1.14	0.22

**Table 3:** Risk for rehospitalization for factors included in the multivariate analysis.

	HR	95% CI Lower	95% CI Upper	*p*-Value
Low risk (reference)				
Intermediate risk	2.11	1.09	4.08	0.02
High risk	0.72	0.33	1.55	0.40
Age	1.01	0.99	1.04	0.08
Gender	1.07	0.81	1.40	0.63

## Data Availability

Data are contained within the article and Supplementary Materials.

## Supplementary Materials

The following supporting information can be downloaded at https://www.mdpi.com/article/10.3390/diseases13100325/s1, Supplementary Table S1. BRASS Scale (Blaylock Risk Assessment Screening Score).
