# Supplementary material for: Revolving Door in Older Patients: An Observational Study of Risk Assessment of Rehospitalization Using the BRASS Scale
Source: Diseases. 2025 Oct 1;13(10):325. doi: 10.3390/diseases13100325 (PMC12562400; doi:10.3390/diseases13100325)
Supplement: Supplementary file 1 [file diseases-13-00325-s001.zip › diseases-3784170-supplementary.pdf]

Supplementary Table S1. BRASS Scale (Blaylock Risk Assessment Screening Score)

Category	Options	Score
**AGE** (choose one)	55 years or younger	0
	56–64 years	1
	65–79 years	2
	80 years or older	3
**LIVING CONDITIONS AND SOCIAL SUPPORT** (choose one)	Lives with spouse	0
	Lives with family	1
	Lives alone with family support	2
	Lives alone with support from friends/acquaintances	3
	Lives alone without any support	4
	Home care/residential care	5
**FUNCTIONAL STATUS** (choose one)	Independent (independent in ADLs and IADLs)	0
	Dependent in:	
	- Feeding/eating	1
	- Bathing/dressing	1
	- Toileting	1
	- Transferring/mobility	1
	- Bowel incontinence	1
	- Urinary incontinence	1
	- Meal preparation	1
	- Medication management	1
	- Managing finances	1
	- Shopping	1

**Category**	**Options**	**Score**
	- Use of transportation	1
**COGNITIVE STATUS** (choose one)	Oriented	0
	Disoriented to some spheres *sometimes	1
	Disoriented to some spheres *always	2
	Disoriented to all spheres *sometimes	3
	Disoriented to all spheres *always	4
	Comatose	5
(*Spheres: place, time, location, and self)		
**BEHAVIORAL MODEL** (choose one)	Appropriate	0
	Wandering (confused)	1
	Agitated	1
	Confused	1
	Other	1
**MOBILITY** (choose one)	Walks independently	0
	Walks with assistive devices	1
	Walks with assistance	2
	Non-ambulatory	3
**SENSORY DEFICITS** (choose one)	None	0
	Visual or hearing impairment	1
	Both visual and hearing impairments	2
**NUMBER OF PREVIOUS HOSPITALIZATION/ EMERGENCY ROOM VISITS** (choose one)	None in the last 3 months	0
	One in the last 3 months	1

Category	Options	Score
	Two in the last 3 months	2
	More than two in the last 3 months	3
**NUMBER OF ACTIVE CLINICAL PROBLEMS** (choose one)	Three clinical problems	0
	Between three and five clinical problems	1
	More than five clinical problems	2
**NUMBER OF MEDICATIONS TAKEN** (choose one)	Fewer than three medications	0
	Between three and five medications	1
	More than five medications	1

Total Score (sum of all points):

Total Score	Risk Index	Description
0–10	Low risk	Patients at low risk of problems after discharge; no special discharge planning needed; disability is very limited.
11–19	Medium risk	Patients at medium risk due to complex clinical situations requiring discharge planning but probably no risk of institutionalization.
≥ 20	High risk	Patients at high risk with significant problems requiring continuity of care, need for rehabilitation or institutional placement.
